# Loss of intestinal sympathetic innervation elicits an innate immune driven colitis

**DOI:** 10.1186/s10020-018-0068-8

**Published:** 2019-01-07

**Authors:** Rose A. Willemze, Olaf Welting, Patricia van Hamersveld, Caroline Verseijden, Laurens E. Nijhuis, Francisca W. Hilbers, Sybren L. Meijer, Balthasar A. Heesters, Joost H. A. Folgering, Harold Darwinkel, Philippe Blancou, Margriet J. Vervoordeldonk, Jurgen Seppen, Sigrid E. M. Heinsbroek, Wouter J. de Jonge

**Affiliations:** 1 Amsterdam UMC, Tytgat Institute for Liver and Intestinal Research, University of Amsterdam, Meibergdreef 69, 1105 BK Amsterdam, The Netherlands; 2 Amsterdam UMC, Department of Pathology, University of Amsterdam, Meibergdreef 9, 1105 AZ Amsterdam, The Netherlands; 3 Amsterdam UMC, Department of Experimental Immunology, University of Amsterdam, Meibergdreef 9, 1105 AZ Amsterdam, The Netherlands; 4 Charles River Laboratories, Discovery, De Mudden 16, 9747 AW Groningen, The Netherlands; 5 Institute of Molecular and Cellular Pharmacology, Nice Sophia Antipolis University, 660 Route des Lucioles, 06560 Valbonne, France; 6 Galvani Bioelectronics, Gunnels Wood Road, Stevenage, Hertfordshire SG1, 2NY UK

**Keywords:** Inflammatory bowel disease, Colitis, Autonomic nervous system, Sympathetic innervation, Norepinephrine

## Abstract

**Background:**

Both the parasympathetic and sympathetic nervous system exert control over innate immune responses. In inflammatory bowel disease, sympathetic innervation in intestinal mucosa is reduced. Our aim was to investigate the role of sympathetic innervation to the intestine on regulation of the innate immune responses.

**Methods:**

In lipopolysaccharide (LPS)-stimulated macrophages, we evaluated the effect of adrenergic receptor activation on cytokine production and metabolic profile. In vivo, the effect of sympathetic denervation on mucosal innate immune responses using 6-hydroxydopamine (6-OHDA), or using surgical transection of the superior mesenteric nerve (sympathectomy) was tested in Rag1^−/−^ mice that lack T- and B-lymphocytes.

**Results:**

In murine macrophages, adrenergic β2 receptor activation elicited a dose-dependent reduction of LPS-induced cytokines, reduced LPS-induced glycolysis and increased maximum respiration. Sympathectomy led to a significantly decreased norepinephrine concentration in intestinal tissue. Within 14 days after sympathectomy, mice developed clinical signs of colitis, colon oedema and excess colonic cytokine production. Both 6-OHDA and sympathectomy led to prominent goblet cell depletion and histological damage of colonic mucosa.

**Conclusions:**

We conclude that the sympathetic nervous system plays a regulatory role in constraining innate immune cell reactivity towards microbial challenges, likely via the adrenergic β2 receptor.

**Electronic supplementary material:**

The online version of this article (10.1186/s10020-018-0068-8) contains supplementary material, which is available to authorized users.

## Background

The intestinal mucosa is home to a dense immune cell network that delicately needs to balance responses to microbiota in the gut lumen. In inflammatory bowel disease (IBD), a complex and multifactorial disease, this crucial balance in the immune system is distorted. Although several therapeutic options are available, not all patients respond to treatment.

Recent trials have taken an original approach for immune suppression in IBD based on the recognition of the nervous system as a negative regulator of inflammatory processes. The vagus nerve has been identified to negatively regulate inflammation in multiple models through the cholinergic anti-inflammatory pathway (Tracey 2002). Vagus nerve stimulation improves colitis in rats (Meregnani et al. 2011). Furthermore, current pilot studies of vagal nerve stimulation are undertaken to support a role for vagus nerve activity in active Crohn’s disease (NCT02311660 and Bonaz et al. (2016)). In addition, earlier studies have put forward that acetylcholine, the main neurotransmitter of the vagus nerve, is a potent immune modulator in gut inflammation (reviewed in (Kawashima et al. 2012)). However, based on anatomical considerations, vagus nerve terminals do not directly innervate mucosal cells nor synapse with mucosal immune cells (Berthoud et al. 1990). Moreover, given the short half-life of acetylcholine, cholinergic modulation of target cells requires close cell-cell interaction and it is unclear whether cholinergic neurons interact with immune cells in the colonic mucosa.

Instead, more recent studies indicate that vagus nerve stimulation reduces inflammation through vagal afferent activity and subsequent activation of the sympathetic greater splanchnic nerves (Martelli et al. 2014; Komegae et al. 2018). The sympathetic nervous system innervates all layers of the intestine and the gut-associated lymphoid tissue (GALT) (Chiocchetti et al. 2008; Kulkarni-Narla et al. 1999). Furthermore, catecholamine levels and sympathetic innervation in the intestine of patients with IBD is decreased (Straub et al. 2008; Magro et al. 2002). Animal models that address the role of adrenergic innervation in colitis have yielded contrasting data. Activating the adrenergic α2 receptor worsened experimental colitis, whereas activation of the adrenergic β3 receptor ameliorates experimental colitis (Vasina et al. 2008; Bai et al. 2009). Furthermore, chemical sympathectomy resulted in a decreased severity of acute experimental colitis but worsened chronic experimental colitis (Straub et al. 2008; McCafferty et al. 1997). We recently established that sympathetic, rather than vagal innervation plays a regulatory role in experimental colitis severity (Willemze et al. 2018). In agreement, previous research has shown that one of the main neurotransmitters of the sympathetic nervous system, norepinephrine, has an anti-inflammatory effect on innate immune cells (Nijhuis et al. 2014; Severn et al. 1992). Norepinephrine can also activate inflammatory cells depending on the binding affinity with different receptors (Straub et al. 2006; Zhou et al. 2005).

The aim of this study was to evaluate potential regulatory role of sympathetic innervation on the level of activation of innate immune cells in the gut mucosa. We show in vitro that adrenergic β2 receptor activation strongly inhibited inflammatory activation by lipopolysaccharide (LPS). In vivo, by using either chemical or surgical sympathectomy in Rag1^−/−^ mice, we show that sympathectomy induces an inflammatory activation of innate immune cells leading to colitis.

## Materials and methods

### Mice

Female C57BL/6 inbred mice (8–12 weeks old) were purchased from Charles River Laboratories (Maastricht, The Netherlands) and male and female Rag1^−/−^ mice (8–12 weeks old) from The Jackson Laboratory (Bar Harbor, ME, USA). Rag1^−/−^Adrβ2^−/−^ mice, male as well as female, were kindly provided to us by the laboratory of Philippe Blancou at the Institute of Molecular and Cellular Pharmacology (Valbonne, France). Animals were housed under specific pathogen free conditions in individually ventilated cages in our animal facility at the Academic Medical Center in Amsterdam. Animals were maintained on a 12/12 h light/dark cycle under constant condition of temperature (20 °C ± 2 °C) and humidity (55%) and ad libitum food and water. Mice were handled in accordance with guidelines of the Animal Research Ethics Committee of the University of Amsterdam. The same ethics committee approved the experimental protocols.

### Murine bone marrow derived macrophages (BMDM)

Bone marrow cells from tibiae and femurs of adult female C57BL/6 mice were cultured for 8 days in RPMI-1640 with HEPES (ThermoFisher Scientific, Landsmeer, The Netherlands) supplemented with 10% (*v*/v) foetal calf serum (FCS; Bodinco, Alkmaar, The Netherlands), 100 U/ml pen/strep (Lonza, Basel, Switzerland), 2 mM L-Glutamine (ThermoFisher Scientific) and 15% (v/v) L929- cell (ATCC, Manassas, VA, USA) conditioned culture medium as a source for macrophage colony-stimulating factor (M-CSF). Cells were harvested with 4 mg/ml lidocaine (Sigma, Zwijndrecht, The Netherlands). BMDM were treated with 100 ng/ml lipopolysaccharide (LPS; Bio-Connect, Huissen, The Netherlands) for 18 h. As a pre-treatment, we used salbutamol, norepinephrine and propranolol (all Sigma) in dosages ranging from 0.1 μM to 10 μM. After 18 h, supernatant was collected for protein determination and BMDM were collected for RNA isolation.

### Human peripheral blood derived macrophages

Peripheral blood mononuclear cells were isolated from healthy donor buffy coats (Sanquin, Amsterdam, The Netherlands) using Ficoll Paque Plus (GE Healthcare, Hoevelaken, The Netherlands) density centrifugation. Subsequently, monocytes were isolated by Percoll (GE Healthcare) density gradient as previously described (Repnik et al. 2003). Cells were cultured at 500,000 cells/ml in a 12-wells plate in RPMI-1640 (ThermoFisher Scientific) and adhered for 1 h and subsequently washed with warm phosphate-buffered saline (PBS). We then added IMDM medium containing HEPES and L-glutamine (Lonza) with 10% (*v*/v) FCS, 100 U/ml pen/strep and 20 ng/ml recombinant human M-CSF (PeproTech, London, UK). Cells were cultured for 8 days to differentiate to macrophages and subsequently treated with 100 ng/ml LPS for 18 h. As a pre-treatment we used salbutamol, norepinephrine and propranolol in dosages ranging from 0.1 μM to 10 μM. After 18 h, we collected supernatant for protein determination and macrophages were collected for RNA isolation.

### Chemical sympathectomy

6-hydroxydopamine (6-OHDA; Sigma), was dissolved in sterile saline containing 0.1% L-ascorbic acid (Sigma) as an antioxidant and was injected intraperitoneally (i.p.) at a concentration of 80 mg/kg body weight on three consecutive days 7 days before the experiment. To maintain sympathectomy, we injected mice i.p. with 6-OHDA (80 mg/kg) every 10 days thereafter. Control animals were injected with saline containing 0.1% L-ascorbic acid as a vehicle.

During the experiment, we recorded bodyweight and behaviour daily. After 28 days, we sacrificed mice and organs were collected. We measured colon wet weights and colon length. Colon weight per 6 cm was used as a disease parameter. Colon tissue was cut in half longitudinally for histopathology. The other half was used for protein analysis.

### Intestine-specific sympathectomy

Sympathetic innervation of the intestine was disrupted by transecting the superior mesenteric nerve along the mesenteric artery (described previously (Willemze et al. 2018; Olivier et al. 2016)). Levels of norepinephrine in ileal tissue after surgery were analysed by mass spectrometry (described previously (Willemze et al. 2018)). The surgical procedure was performed on anesthetized animals under 2% isoflurane/O_2_. Pre-operatively, animals were treated with Metacam 5 mg/kg (Boehringer, Ingelheim am Rein, Germany).

Following sympathectomy or sham laparotomy, we recorded bodyweight. After 2 weeks, just before sacrifice, we rated colonic inflammation by endoscopy of the colon under anaesthesia with 3% isoflurane/O_2_. The Olympus URF type V endoscope (Zoeterwoude, The Netherlands) was rectally inserted for a maximum of 5 cm and videos of the endoscopy were recorded using a Medicap USB200 Medical Digital Video Recorder (Roermond, The Netherlands), when retracting the endoscope. A blinded and trained technician determined the adjusted murine endoscopic index of colitis severity (MEICS), consisting of wall thickening, vascularity, visible fibrin and granularity (Becker et al. 2005). Wall thickening grade 0 - transparent; 1 - moderate; 2 - marked; 3 - intransparent. Vascularity grade 0 - normal; 1 - moderate; 2 - marked; 3 - bleeding. Visible fibrin grade 0 - none; 1 - little; 2 - marked; 3 - extreme. Granularity score 0 - none; 1 - moderate; 2 - marked; 3 - extreme. This determined a total endoscopy score ranging from 0 to 12. After endoscopy, animals were sacrificed and organs collected. We measured colon wet weights and colon length. Colon weight per 6 cm was used as a disease parameter. Colon tissue was cut in half longitudinally for histopathology. The other half of the colon and the last 5 cm of the ileum were used for quantitative PCR analysis.

### Isolation and sorting of colonic lamina propria cells

To isolate single cell populations with fluorescence activated cell sorting, a follow up sympathectomy experiment was performed. After 2 weeks animals were sacrificed and colons were cut open longitudinally and cut in 0.5 cm pieces, washed with PBS and incubated in calcium/magnesium-free Hanks’ balanced salt solution (HBSS; Lonza) supplemented with 2% (*v*/v) FCS containing 5 mM EDTA (ThermoFisher Scientific) for 20 min, shaking at 230 rpm at 37 °C. After incubation, tissue was washed with PBS and cut into 0.1 cm pieces. Tissue pieces were incubated for 45 min under vigorous shaking at 37 °C with calcium/magnesium-free HBSS supplemented with 2% FCS, 62.5 μg/ml Liberase TL (Roche Applied Bioscience, Almere, The Netherlands; Cat No 05401020001) and 200 μg/ml DNase I (Roche Applied Bioscience; Cat No 11284932001). To obtain a single cell suspension, we passed the suspension through a 100 μm cell strainer after complete digestion. Cells were stained for 30 min on ice in PBS containing 0.5% bovine serum albumin (BSA; Sigma) and 2 mM EDTA with the following specific antibodies: APC-Cy7 –conjugated anti-CD45 (30-F11; Biolegend, San Diego, CA, USA), DAPI (Brunschwig, Amsterdam, The Netherlands), PerCP-conjugated CD11b (M1/70; Biolegend), FITC-conjugated Ly6G (1A8; Biolegend), PE-conjugated CD64 (X54–5/7.1; Biolegend), Alexa700-conjugated CD11c (N418; Affymetrix, Vienna, Austria), APC-conjugated Ly6C (HK1.4; Affymetrix) and Pe-Cy7-conjugated MHC-II (AF6–120.1; Biolegend). Cells were sorted using a FACS Aria cell sorter (BD Bioscience, San Jose, CA, USA) and collected in RPMI 1640 with 40 U/ml Ribolock (ThermoFisher Scientific).

### RNA isolation, cDNA synthesis and quantitative PCR analysis

RNA was isolated from murine BMDM and human macrophages with the Bioline ISOLATE II mini kit (GC biotech B.V., Alphen a/d Rijn, The Netherlands) according to manufacturer’s protocol. We extracted RNA from snap-frozen ileal and colonic tissue after homogenization of the samples in TriPure isolation reagent according to manufacturer’s instructions (Roche Applied Science). RNA isolation from sorted colonic lamina propria cells was performed with the Bioline ISOLATE II micro kit (GC biotech B.V.) following manufacturer’s instructions.

cDNA was synthesized using dNTPs (ThermoFisher Scientific), Random primers (Promega, Leiden, The Netherlands), Oligo dT primers (Sigma), Revertaid and Ribolock. Quantitative polymerase chain reaction (PCR) was performed using SensiFAST SYBR No-ROX (GC biotech B.V.) on a LightCycler 480 II (Roche Applied Science) to analyse expression levels of murine interleukin (IL)-1β, IL-6, IL-10, IL-12, IL-22, tumour necrosis factor (TNF)-α, nitric oxide synthase 2 (Nos2), arginase 1 (Arg1), regenerating islet-derived protein 3γ (Reg3γ), defensin α (DefA) and mucin 2 (Muc2) using LinRegPCR software (Ruijter et al. 2009). For normalization murine reference genes β-actin, ubiquitin, hypoxanthine phosphoribosyltransferase (HPRT), cyclophilin and glyceraldehyde-3-phosphate dehydrogenase (GAPDH) were selected, after analysis for stability in geNorm (Vandesompele et al. 2002). In human macrophages, expression levels of human IL-1β, IL-6, IL-10, IL-12, TNF-α were measured and normalized using human reference genes β-actin and β2 microglobulin, selected after analysis for stability in geNorm. Primers (synthesized by Sigma) are listed in Table 1 (murine) and Table 2 (human).

**Table 1:** Mouse primers

Gene	Forward sequence	Reverse sequence
IL-1β	GCCCATCCTCTGTGACTCAT	AGGCCACAGGTATTTTGTCG
IL-6	GAGTTGTGCAATGGCAATTCTG	TGGTAGCATCCATCATTTCTTTGT
IL-10	TGTCAAATTCATTCATGGCCT	ATCGATTTCTCCCCTGTGAA
IL-12	AGACCCTGCCCATTGAACTG	CGGGTCTGGTTTGATGATGTC
TNF-α	TGGAACTGGCAGAAGAGGCACT	CCATAGAACTGATGAGAGGGAGGC
Nos2	TTCTGTGCTGTCCCAGTGAG	TGAAGAAAACCCCTTGTGCT
Arg1	CTCCAAGCCAAAGTCCTTAGAG	AGGAGCTGTCATTAGGGACATC
IL-22	CGGCTCATCGGGGAGAAAC	TGACTGGGGGAGCAGAACG
Reg3γ	TCCACCTCTGTTGGGTTCAT	AAGCTTCCTTCCTGTCCTCC
DefA	CGCAGCCATGAAGAAACTTG	GAATCAGCCTGGACCTGGAA
Muc2	TGAAGACCGAGATTGTGCCC	AGATGACGTTGAGCTGGGTG
β-actin	TTCTTTGCAGCTCCTTCGTT	ATGGAGGGGAATACAGCCC
Ubiquitin	AGCCCAGTGTTACCACCAAG	ACCCAAGAACAAGCACAAGG
HPRT	CCTAAGATGAGCGCAAGTTGAA	CCACAGGACTAGAACACCTGCTAA
Cyclophilin	ATGGTCAACCCCACCGTGT	TTCTGCTGTCTTTGGAACTTTGTC
GAPDH	ATGTGTCCGTCGTGGATCTGA	ATGCCTGCTTCACCACCTTCT
RPLP0	CCAGCGAGGCCACACTGCTG	ACACTGGCCACGTTGCGGAC

**Table 2:** Human primers

Gene	Forward sequence	Reverse sequence
IL-1β	GAAGCTGATGGCCCTAAACA	AAGCCCTTGCTGTAGTGGTG
IL-6	AGTGAGGAACAAGCCAGAGC	GTCAGGGGTGGTTATTGCAT
IL-10	GCCACCCTGATGTCTCAGTT	GTGGAGCAGGTGAAGAATGC
IL-12	ATGCCTTCACCACTCCCAAA	TAGAGTTTGTCTGGCCTTCTGG
TNF-α	CCTGCTGCACTTTGGAGTGA	GAGGGTTTGCTACAACATGGG
β-actin	AGAGCTACGAGCTGCCTGAC	AGCACTGTGTTGGCGTACAG
β2 microglobulin	CTCGCGCTACTCTCTCTCTTTCT	TGCTCCACTTTTTCAATTCTCT

### Measurement of protein concentrations of cytokines

Snap-frozen colonic tissue was homogenized on ice in Greenberger Lysis Buffer (150 mM NaCl, 15 mM Tris, 1 mM MgCl·6H2O, 1 mM CaCl2, 1% Triton) with protease inhibitor cocktail (Roche Applied Science; Cat No 11697498001), pH 7.4, diluted 1:1 with PBS.

In supernatant of BMDM and colonic tissue, concentrations were determined of IL-1β, IL-4, IL-6 IL-10, IL-17, interferon (IFN)-γ and TNF-α by sandwich enzyme-linked immunosorbent assay (ELISA; R&D systems, Abingdon, UK) according to manufacturer’s protocol. Values were normalized using total protein levels measured with the Pierce BCA Protein Assay Kit (ThermoFisher Scientific) following manufacturer’s protocol. In supernatants of human macrophages, protein concentrations of IL-1β, IL-6, IL-10, IL-12 and TNF-α were measured with a human inflammation kit by BD Cytometric Bead Assay (CBA; BD Bioscience) according to manufacturer’s protocol, with the exception that reagents were 10 times diluted.

### Extracellular flux assay measuring energy metabolism

BMDM were plated in a Seahorse 96-well XF plate (Agilent, Santa Clara, CA, USA) and adhered for 1 h at room temperature and then equilibrated at 37 °C. Three hours after plating, BMDM were treated with 100 ng/ml LPS for 18 h. As a pre-treatment, we used salbutamol, norepinephrine and propranolol in dosages ranging from 0.1 μM to 10 μM. Experiment was adapted from a previously published protocol (Van den Bossche et al. 2015; de Moura and Van Houten 2014). In short, oxidative phosphorylation in BMDM was measured by the Oxygen Consumption Rate (OCR, pmol/min) and glycolysis was measured by Extracellular Acidification Rate (ECAR, pmol/min). Glucose, oligomycin, trifluoromethoxy carbonylcyanide phenylhydrazone (FCCP), rotenone and antimycin A (all Sigma) were used to measure OCR and ECAR. We used Seahorse XFe96 Analyzer (Agilent Technologies, Amstelveen, the Netherlands) for the assay and Wave Desktop Software version 2.3 (Agilent) to calculate the OCR and ECAR.

### Histopathology

Colon tissue was fixed in 4% formalin according to protocol and embedded in paraffin for routine histology. A blinded and experienced pathologist evaluated formalin-fixed haematoxylin and eosin (HE) stained tissue sections microscopically and scored the sections based on seven characteristics of inflammation explained in Table 3. This resulted in a total histology score ranging from 0 to 23.

**Table 3:** Histology score adapted from Read et al. (2001)

Score	0	1	2	3	4
Mono- and polymorphonuclear infiltrate	Normal	Increase in mucosa	Increase in mucosa and submucosa	Extending into mucosa, submucosa, tunica muscularis and/or serosa	
Goblet cells	Normal, large amount	Depletion of < 10%	Depletion of 10–50%	Depletion of > 50%	
Crypt loss	No crypt loss	< 10% crypt loss	10–50% crypt loss	> 50% crypt loss	
Epithelial hyperplasia	Normal	Slight hyperplasia	2-3x increase of crypt length	>3x increase of crypt length	
Ulcerations	No ulceration				Ulcerations
Crypt abscesses	No abscesses				Crypt abscesses

### Statistical analysis

Graphs were made with Prism 7.0 (GraphPad Software, La Jolla, CA). For all experiments, we used a Kolmogorov-Smirnov test to determine normality of distribution. In IBM SPSS Statistics Version 23 (IBM Corporation, Armonk, NY), an independent T-test or Mann-Whitney U test (2 groups) or an ANOVA or Kruskal-Wallis test (> 2 groups) was used to check for statistical significance. When the ANOVA analysis gave a significant difference, then a Bonferroni correction for multiple comparisons was done. When the Kruskal-Wallis analysis gave a significant difference, a pairwise comparison with post-hoc Dunn’s test was done. All data are expressed as mean (if distributed normally) or median (if not distributed normally) plus standard deviation (SD), interquartile range (IQR) or individual data points. *P*-value (p) < 0.05 was considered significant.

## Results

### Adrenergic receptor activation reduces LPS-induced inflammatory responses in macrophages

**Fig. 1:**
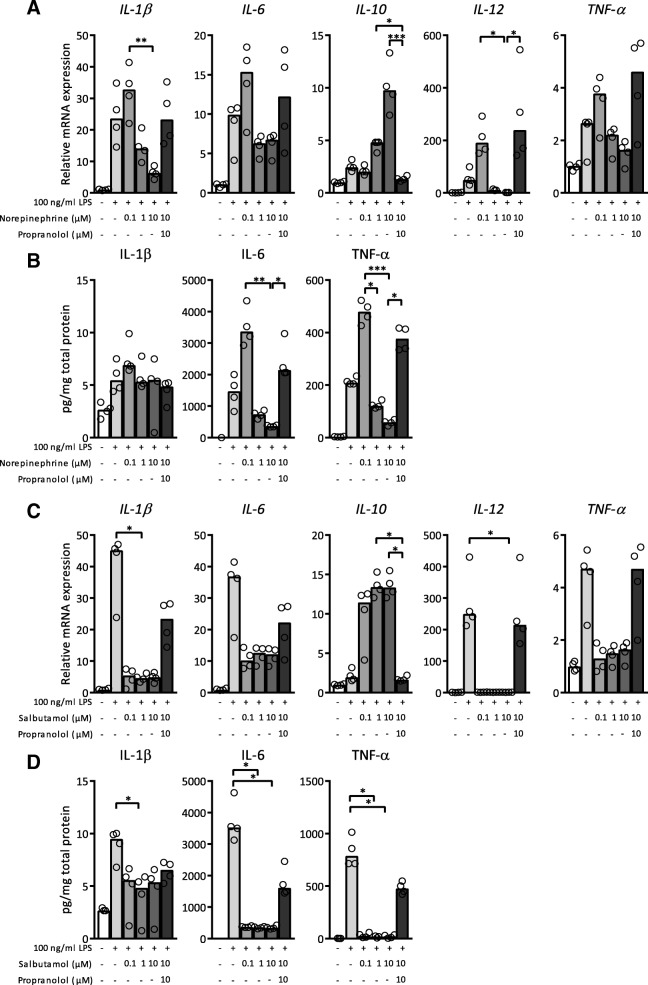
Lipopolysaccharide (LPS)-exposed bone marrow derived macrophages (BMDM) produced less pro-inflammatory cytokines after pre-treatment with norepinephrine or salbutamol. (A&C) mRNA expression levels of interleukin (IL)-1β, IL-6, IL-10, IL-12 and tumour necrosis factor (TNF)-α in cell lysate of BMDM after treatment with 100 ng/ml LPS for 18 h and pre-treatment with different concentrations of norepinephrine (**a**) or salbutamol (**c**) together with propranolol, normalized for reference genes β-actin and Ubiquitin. Expression is relative to mRNA expression in BMDM not exposed to any treatment. (B&D) Protein levels of IL-1β, IL-6 and TNF-α in supernatant of BMDM after treatment with 100 ng/ml LPS for 18 h and pre-treatment with different concentrations of norepinephrine (**b**) or salbutamol (**d**) together with propranolol, normalized for total protein levels. *N* = 4 mice. Data is expressed as median and individual data points. We tested for statistical significant differences with a Kruskal-Wallis test and post-hoc Dunn’s test. *P*-value < 0.05 was considered significant. * *P*-value < 0.05; ** *P*-value ≤0.01; *** *P*-value ≤0.001

We first investigated the effect of adrenergic receptor activation on LPS-activated bone marrow derived macrophages (BMDM). BMDM were pre-treated with different dosages of norepinephrine and subsequently stimulated with 100 ng/ml LPS. The lowest concentration (0.1 μM) of norepinephrine increased LPS-induced mRNA expression levels of IL-1β, IL-6, IL-12 and TNF-α whereas higher norepinephrine concentrations significantly decreased cytokine expression and release (Fig. 1a, b). For the anti-inflammatory cytokine IL-10, the opposite effect was observed. In absence of LPS stimulation, norepinephrine did not alter mRNA expression (data not shown). Propranolol, a non-selective beta-blocker, reversed effects elicited by norepinephrine showing that effects on cytokine release through norepinephrine mainly involve adrenergic β receptors.

**Fig. 2:**
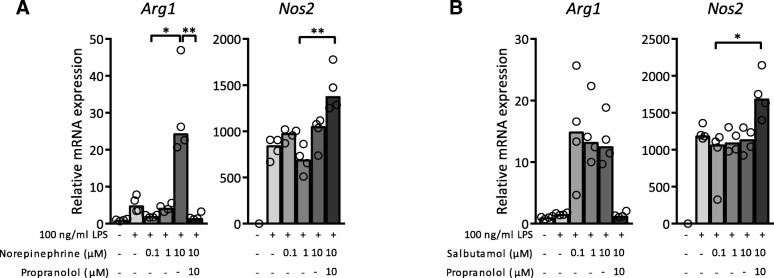
Lipopolysaccharide (LPS)-exposed bone marrow derived macrophages (BMDM) had an increased anti-inflammatory phenotype after adrenergic receptor activation. mRNA expression levels of Arginase 1 (Arg1) and nitric oxide synthase 2 (Nos2) in cell lysate of BMDM after treatment with 100 ng/ml LPS for 18 h and pre-treatment with different concentrations of norepinephrine (**a**) or salbutamol (**b**) together with propranolol, normalized for reference genes β-actin and Ubiquitin. Expression is relative to mRNA expression in BMDM not exposed to any treatment. *N* = 4 mice. Data is expressed as median and individual data points. We tested for statistical significant differences with a Kruskal-Wallis test and post-hoc Dunn’s test. *P*-value < 0.05 was considered significant. * *P*-value < 0.05; ** *P*-value ≤0.01

To specify the role of the adrenergic β2 receptor, LPS-exposed BMDM were pre-treated with salbutamol, a selective adrenergic β2 receptor agonist. Salbutamol dose-dependently reduced LPS-induced mRNA expression of IL-1β, IL-6, IL-12 and TNF-α, and elevated IL-10 expression (Fig. 1c). In absence of LPS stimulation, salbutamol did not alter mRNA expression (data not shown). Propranolol blocked the effect of salbutamol. In agreement with transcriptional data, LPS-elicited protein levels of IL-1β, IL-6 and TNF-α were reduced by salbutamol pre-treatment (Fig. 1d). Complimentary to data shown in Fig. 1, Arg1, a marker for anti-inflammatory, M2 polarized macrophages, (Wynn et al. 2013) was upregulated after pre-treatment with 10 μM norepinephrine and salbutamol compared to vehicle control (Fig. 2). Interestingly, propranolol further increased expression of LPS-induced Nos2, a marker for pro-inflammatory, M1 polarized macrophages, (Wynn et al. 2013) whereas activation of β2 adrenergic receptors did not lead to decreased Nos2 mRNA expression (Fig. 2).

We next analysed human peripheral blood derived macrophages in a similar setup. A significant difference was reached for IL-6 mRNA expression comparing 1 μM norepinephrine pre-treatment to norepinephrine combined with propranolol as a pre-treatment (resp. median(IQR): 0.8(0.1) vs. 1.5(0.2); *p* = 0.025; Additional file 1: Figure S1A). However, in contrast to mouse cells, LPS-exposed human macrophages showed no significant reduction of cytokines production after pre-treatment with salbutamol (Additional file 1: Figure S1C-D).

### Adrenergic receptor activation affects the metabolic profile of LPS-stimulated macrophages

**Fig. 3:**
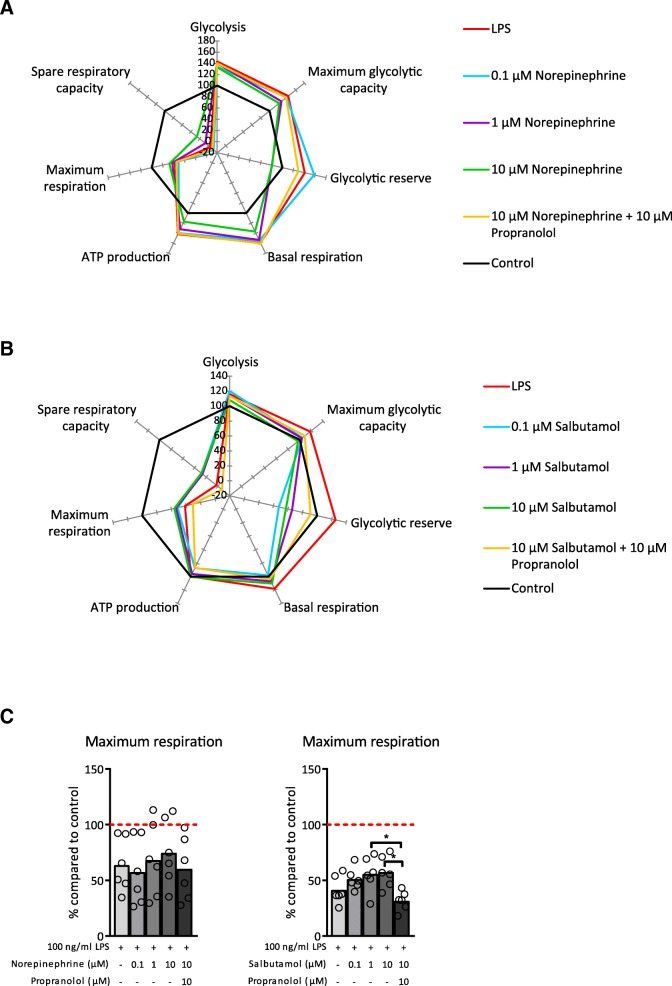
Pre-treatment with norepinephrine or salbutamol altered the metabolic profile of lipopolysaccharide (LPS)-exposed bone marrow derived macrophages (BMDM). **a**, **b** Metabolic parameters (described in van den Bossche et al. (2015)) of BMDM after treatment with 100 ng/ml LPS for 18 h and pre-treatment with different concentrations of norepinephrine (**a**) or salbutamol (**b**) together with propranolol. Data is shown relative to BMDM not exposed to any treatment (control). **c** Maximum respiration of BMDM after treatment with 100 ng/ml LPS for 18 h and pre-treatment with different concentrations of norepinephrine or salbutamol together with propranolol. Data is shown relative to BMDM not exposed to any treatment (red dashed line). *N* = 6 mice. Data is expressed as mean and individual data points. We tested for statistical significant differences with an ANOVA and post-hoc Bonferroni correction. *P*-value < 0.05 was considered significant. * *P*-value < 0.05

Macrophage activation elicits changes in their metabolic profile according to their activation state. It has been shown that LPS-exposed BMDM adopt a glycolytic metabolic profile, whereas IL-4 stimulation elicits an oxidative phosphorylation driven metabolic profile (Van den Bossche et al. 2015). To investigate the effect of adrenergic receptor activation on LPS-induced glycolytic metabolic profile of BMDM, we made use of an extracellular flux assay. Pre-treatment with norepinephrine and salbutamol inhibited LPS-induced glycolysis and led to a metabolic profile indicating higher oxidative phosphorylation. Propranolol pre-treatment reversed the effect of norepinephrine (Fig. 3) demonstrating that these effects were mediated through adrenergic β receptors. Salbutamol treatment showed similar results as norepinephrine indicating that signalling via the adrenergic β2 receptor caused the change in metabolic profile.

### Treatment with 6-OHDA induces histologic abnormalities in Rag1^−/−^ mice

**Fig. 4:**
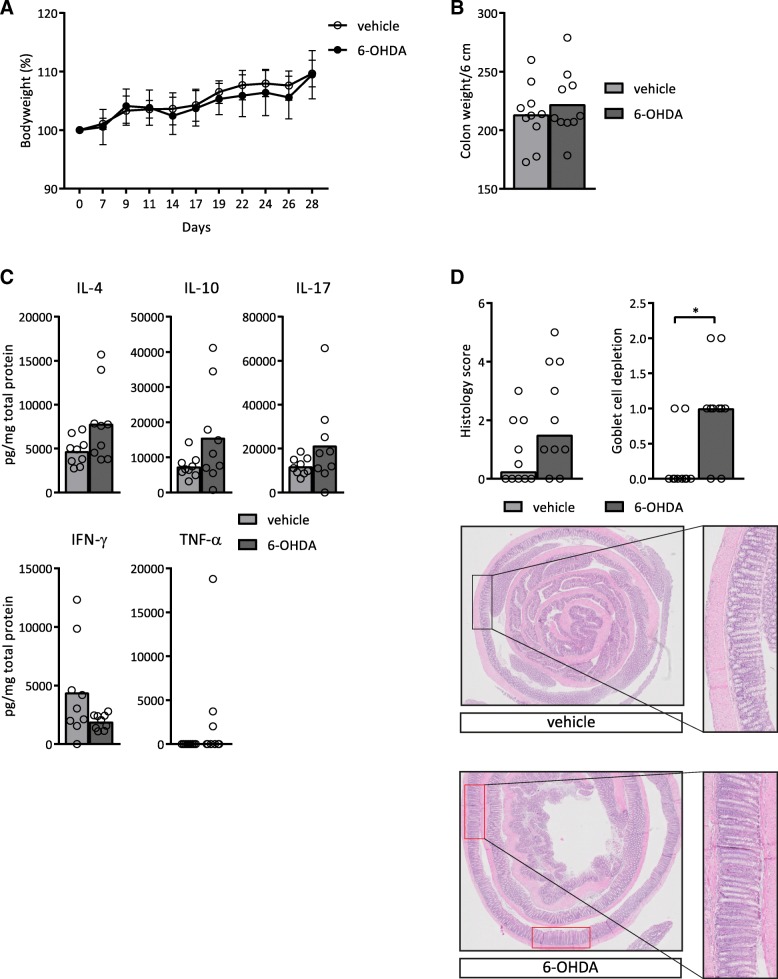
Rag1^−/−^ mice showed a higher histology score after treatment with 6-OHDA. **a** Bodyweight of mice over time. **b** Colon weight as a marker of inflammation, normalized for colon length. **c** Protein levels of interleukin (IL)-4, IL-10, IL-17, interferon (IFN)-γ and tumour necrosis factor (TNF)-α in colon homogenates, normalized for total protein levels. **d** Total histology score as described in Table 3 and goblet cell depletion subscore. Representative pictures are shown of a haematoxylin and eosin (HE) staining of the colon. 2x magnification. The areas within the red boxes are examples of goblet cell depletion. The black box shows an area with normal goblet cell numbers. An enlargement is shown to the right. For more examples and details of the histology score, see also Additional file 2: Figure S2. *N* = 10 mice. Data is expressed as mean (**a**, **b** and **c**) or median (TNF-α from **c** and **d**) and individual data points (all except A where standard deviation is shown). We tested for statistical significant differences with an independent T-test or a Mann-Whitney U test. *P*-value < 0.05 was considered significant. * *P*-value < 0.05

To assess the impact of the sympathetic nervous system in vivo, we made use of treatment with the catecholaminergic neurotoxin 6-OHDA, which causes chemical sympathetic denervation if systemically administered (Glinka et al. 1997). To be able to study the influence of the sympathetic denervation on myeloid immune cells, we used Rag1^−/−^ mice, lacking T- and B-lymphocytes. No differences were observed in bodyweight loss over time, colon weight or colonic cytokine levels between Rag1^−/−^ mice treated with 6-OHDA and Rag1^−/−^ mice without 6-OHDA treatment (Fig. 4a-c). However, 6-OHDA led to histological features of colitis and the subscore ‘goblet cell depletion’ was significantly elevated in mice treated with 6-OHDA (median(IQR): 1(0.5)) compared to the mice without treatment with 6-OHDA (median(IQR): 0(0.3); *p* = 0.015; Fig. 4d). These results indicate that chemical sympathetic denervation decreases the number of goblet cells in the intestinal epithelium, which is also a known histological feature of colitis.

### Rag1^−/−^ mice develop colitis after intestine-specific sympathectomy

**Fig. 5:**
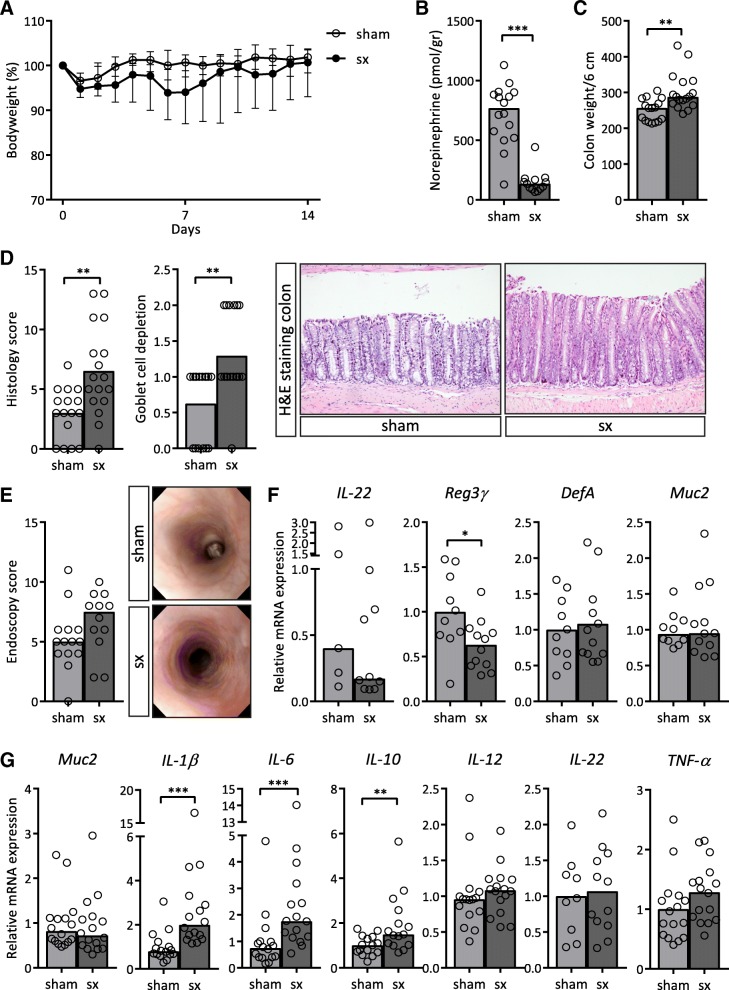
Rag1^−/−^ mice developed colitis after intestine-specific sympathectomy. **a** Bodyweight of mice over time, comparing mice after sympathectomy (sx) to mice after a sham operation (sham). **b** Norepinephrine levels in ileum normalized per gram tissue. **c** Colon weight as a marker of inflammation, normalized for colon length. **d** Total histology scores as described in Table 3 and goblet cell depletion subscore. Representative pictures are shown of a haematoxylin and eosin (HE) staining of the colon. 10x magnification. **e** Total endoscopy score at day 14. Representative pictures are shown. **f** mRNA expression levels in ileum homogenates of interleukin (IL)-22, regenerating islet-derived protein 3 γ (Reg3γ), defensin α (DefA) and mucin 2 (Muc2). **g** mRNA expression levels of goblet cell marker Muc2 and cytokines IL-1β, IL-6, IL-10, IL-12, IL-22 and tumour necrosis factor (TNF)-α in colon homogenate. **f**, **g** Expression in ileum was normalized for reference genes β-actin and hypoxanthine phosphoribosyltransferase (HPRT). Expression in colon was normalized for reference genes ubiquitin and cyclophilin. Expression is relative to the sham group. *N* = 10–16 mice. Data is expressed as mean (**d**, Reg3γ and DefA from **f** and IL-22 and TNF-α from **g**) or median (**a**-**c**, **e**, IL-22 and Muc2 from **f** and **g**) and individual data points or interquartile range is shown. We tested for statistical significant differences with an independent T-test or a Mann-Whitney U test. *P*-value < 0.05 was considered significant. * *P*-value < 0.05; ** *P*-value ≤0.01; *** *P*-value ≤0.001

As 6-OHDA is an established method to achieve sympathetic dysfunction, it is not selective and may not cause complete denervation. To achieve the latter, we next performed surgical sympathectomy (or a control laparotomy (sham)) by cutting the superior mesenteric nerve supplying sympathetic innervation to the intestine. As expected, after surgery a drop in bodyweight was observed in both operated groups due to surgery. Mice in the sham group quickly recovered from this initial weight loss. In contrast, mice in the sympathectomy group did not gain weight over a time period of 12 days (Fig. 5a). Ileal norepinephrine levels in the sympathectomy group (median(IQR): 136(83.45) pmol/gr) were significantly lower compared to the sham group (median(IQR): 768.5(362.7) pmol/gr; *p* < 0.001; Fig. 5b). Colon weights were significantly higher in the sympathectomy group (median(IQR): 288(64.1) mg/6 cm) compared to the sham group (median(IQR): 256.7(60.6) mg/6 cm; *p* = 0.002; Fig. 5c). Total histology score for colitis was significantly higher in the sympathectomy group (mean(SD): 6.5(3.7)) compared to the sham group (mean(SD): 3(2.1); p = 0.002; Fig. 5d; Additional file 2: Figure S2). In addition to inflammatory parameters, mRNA expression of antimicrobial peptide Reg3γ was significantly decreased in the ileum of the sympathectomy group (mean(SD): 0.6(0.3)) compared to the sham group (mean(SD): 1.0(0.4); *p* = 0.03) whilst other genes involved in mucosal antimicrobial defence were not affected (Fig. 5f). In the colon, transcript levels of IL-1β, IL-6 and IL-10 were significantly higher after sympathectomy compared to sham (IL-1β median(IQR): 2.0(1.8) vs. 0.8(0.5); *p* < 0.0001; IL-6 median(IQR): 1.8(1.9) vs. 0.7(0.6); *p* = 0.001; IL-10 median(IQR): 1.5(1.6) vs. 1.0(0.7); *p* = 0.009; Fig. 5g).

Considering this upregulation of colonic cytokines after sympathectomy and the effect of norepinephrine on murine macrophages (Fig. 1), the colitis seen after sympathectomy is likely due to enhanced reactivity of monocyte and macrophage cell populations to luminal microbiota. In vitro, adrenergic receptor activation reduced LPS-induced inflammatory responses in BMDM from Rag1^−/−^ mice, similar to BMDM derived from C57Bl/6 mice (Additional file 3: Figure S3A). However, Rag1^−/−^ mice that also lack the adrenergic β2 receptor (Rag1^−/−^Adrβ2^−/−^) showed no reduced expression of IL-6, IL-12 and TNF-α and no increased expression of IL-10 and Arg1 after pre-treatment of norepinephrine or salbutamol (Additional file 3: Figure S3B). This underlines the critical importance of the adrenergic β2 receptor in reducing LPS-induced inflammatory responses.

**Fig. 6:**
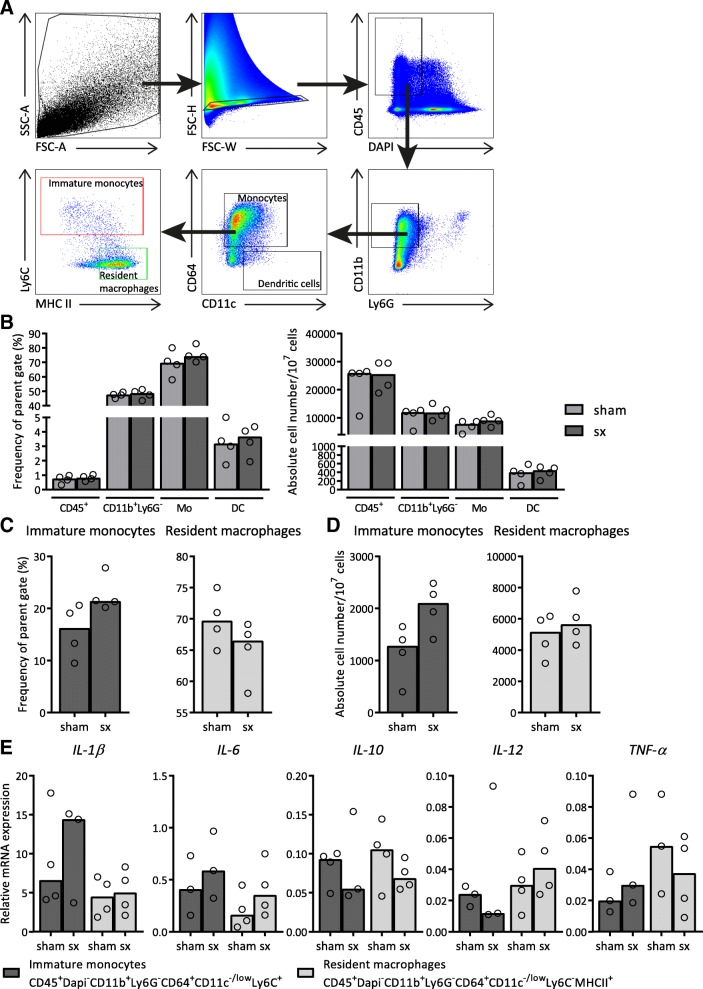
The colon myeloid immune compartment of Rag1^−/−^ shifted towards a more pro-inflammatory phenotype after intestine-specific sympathectomy. **a** Gating strategy. **b** Frequency of parent (%) and absolute cell number of different myeloid populations (CD45^+^: CD45^+^ cells, Mo: monocytes, DC: dendritic cells) in the colon of Rag1^−/−^ mice 2 weeks after sympathectomy (sx) or a sham operation (sham). **c** Frequency of parent (%) of immature monocytes and resident macrophages present in the colon. **d** Absolute cell numbers of immature monocytes and resident macrophages present in the colon. **e** mRNA expression levels of interleukin (IL)-1β, IL-6, IL-10, IL-12 and tumour necrosis factor (TNF)-α in sorted immature monocytes and resident macrophages from the colon. We normalized expression for reference genes β-actin and glyceraldehyde-3-phosphate dehydrogenase (GAPDH). *N* = 10–11 mice pooled per 2–3 animals for analysis. Data is expressed as median and individual data points. We tested for statistical significant differences with a Mann-Whitney U test. *P*-value < 0.05 was considered significant

To determine the effect of sympathectomy on different colonic myeloid subsets, we isolated these cells after sympathectomy or sham surgery in Rag1^−/−^ mice. We regarded CD11b^+^Ly6G^−^CD64^+^CD11c^-/low^Ly6C^+^ cells as immature monocytes and CD11b^+^Ly6G^−^CD64^+^CD11c^-/low^Ly6C^+^MHCII^high^ cells as resident macrophages in the colon (Fig. 6a), adapted from Bain et al. (2014; 2013). We found no difference in the total innate immune compartment in the mucosa between the sympathectomy and the sham group (Fig. 6b). Further analyses suggested an elevated frequency and absolute cell number of immature monocytes after sympathectomy, possibly reflecting a colitic state of the tissue (Fig. 6c, d). We next analysed mRNA expression of these two subsets and found that both subsets had higher mRNA expression levels of pro-inflammatory cytokines and lower mRNA expression levels of IL-10 in the sympathectomy group compared to the sham group (Fig. 6e). However, this did not reach statistical significance. Irrespective, our results imply that intact sympathetic innervation of the colon is required in regulation of the intestine myeloid compartment in Rag1^−/−^ mice.

## Discussion

Regulation of immune responses has long been viewed as autonomous, mediated by interactions between immune cells in a largely self-regulated system. However, more recent research has provided substantial evidence that in this balance, the nervous system has an active regulatory function (Bellinger and Lorton 2014). Over the last decades, the vagus nerve has been extensively studied for its potential in IBD treatment (Meregnani et al. 2011; Bonaz et al. 2016). We recently demonstrated that for experimental IBD models, sympathetic, rather than vagal, innervation to the gut plays a critical role in maintaining immune homeostasis (Willemze et al. 2018). Here, we show that adrenergic receptor activation has a potent anti-inflammatory effect on murine macrophages and that abrogation of sympathetic innervation to the intestine causes innate immune driven colitis in Rag1^−/−^ mice.

LPS-exposed murine BMDM produced lower mRNA and protein levels of pro-inflammatory cytokines after adrenergic receptor stimulation. This effect is still present 18 h after washout of adrenergic receptor agonists, which indicates a stable effect. These findings are in line with previous data that showed that dendritic cell tolerogenic capacity increases after stimulation with adrenergic receptor agonists (Nijhuis et al. 2014). Salbutamol, an adrenergic β2 receptor selective agonist, caused similar or even stronger effects as compared to norepinephrine indicating that the adrenergic β2 receptor is the main receptor responsible for the anti-inflammatory effect. Salbutamol is a very strong agonist and more potent than norepinephrine for activating the adrenergic β2 receptor, which might explain differences in effect between norepinephrine and salbutamol. Furthermore, norepinephrine binds to all adrenergic receptors potentially causing opposing effects than solely adrenergic β2 receptor activation with salbutamol. It should be emphasized that there are different classes of adrenergic receptors, which elicit distinct biological responses upon binding. Likewise, many immune cells express adrenergic receptors but the eventual outcome of receptor ligation differs depending on norepinephrine concentration, expression of adrenergic receptor subclass, and also on the time point of sympathetic nervous system activation in relation to immune responses (Straub et al. 2006). For instance, adrenergic α2 receptor activation leads to progression of acute colitis, whereas adrenergic β3 receptor activation ameliorated experimental colitis (Vasina et al. 2008; Bai et al. 2009). Also in our in vitro setting, lower levels of norepinephrine led to more pro-inflammatory state, but higher concentrations resulted in the opposite, an anti-inflammatory state. This is likely explained by specific binding affinities for the receptors at different concentrations and brings into perspective the decreased sympathetic innervation, and subsequent norepinephrine level, in IBD patients (Straub et al. 2008; Magro et al. 2002). This is exemplified by our observations on the effects of different concentrations of norepinephrine on cytokine production. Expression of anti-inflammatory cytokine IL-10 is increased at norepinephrine concentrations of 10 μM, whereas pro-inflammatory cytokines as TNF-α are already increased by lower concentrations.

It is known that macrophages exhibit a different metabolic profile depending on their activation state (Van den Bossche et al. 2015). In addition to this, there is also evidence that Krebs cycle intermediates function as immune signalling molecules (Williams and O’Neill 2018). Because we show that norepinephrine affects the metabolic state of macrophages, our results indicate the importance of adrenergic stimulation for pro- and anti-inflammatory properties of these cells.

We observed no pronounced anti-inflammatory effect of adrenergic receptor stimulation in human macrophages. However, it has been shown that adrenergic β receptor signalling lowers TNF-α production of whole blood treated with LPS (Severn et al. 1992; Hong et al. 2015). Furthermore, in LPS-exposed human monocytes, adrenergic α1 receptor activation is shown to reduce IL-1β production (Horstmann et al. 2016). In addition to this, in different human macrophage cell lines, the effect of norepinephrine on pro-inflammatory cytokine production is inconsistent (Severn et al. 1992; Li et al. 2015). These in vitro studies were not performed in differentiated primary human macrophages making comparison with our results difficult. An explanation might be that human macrophages desensitise to adrenergic receptor activation. However, the importance of our findings in human colitis has to be established.

Our results complement a recent study showing that macrophages in the intestinal muscularis polarize towards a more anti-inflammatory, tissue-protective macrophage after adrenergic β2 receptor activation (Gabanyi et al. 2016). Additionally, our work is in line with increasing recognition that the sympathetic nervous system is crucial to regulate the immune response targeting lymphoid organs. Earlier work showed that the anti-inflammatory effect of the vagus nerve depends on the spleen and in its sympathetic innervation (Huston et al. 2006; Rosas-Ballina et al. 2008; Nance and Sanders 2007; Bratton et al. 2012). Furthermore, it is suggested that the sympathetic splanchnic nerve is necessary for the cholinergic anti-inflammatory pathway and interaction with the spleen (Martelli et al. 2014). Another study showed that the sympathetic nervous system controls lymphocyte egress from lymph nodes and numbers of circulating lymphocytes (Nakai et al. 2014). Furthermore, catecholamines increase migration and proliferation potential of myeloid precursor cells from bone marrow (Spiegel et al. 2007).

As our main interest was the innate immune response, we made use of Rag1^−/−^ mice to evaluate the effect of 6-OHDA or surgical sympathectomy. Our data indicate that in both models, sympathetic denervation causes some degree of colitis without an additional trigger of inflammation, with one of the major characteristics being loss of goblet cells. It needs to be emphasized that Rag1^−/−^ mice have other immune cells besides myeloid cells like natural killer cells and innate lymphoid cells that we did not investigate. However, since norepinephrine has a very potent effect in vitro on macrophages and that denervation in vivo leads to alterations in macrophages it is likely that these cells are critical in maintaining immune homeostasis.

**Fig. 7:**
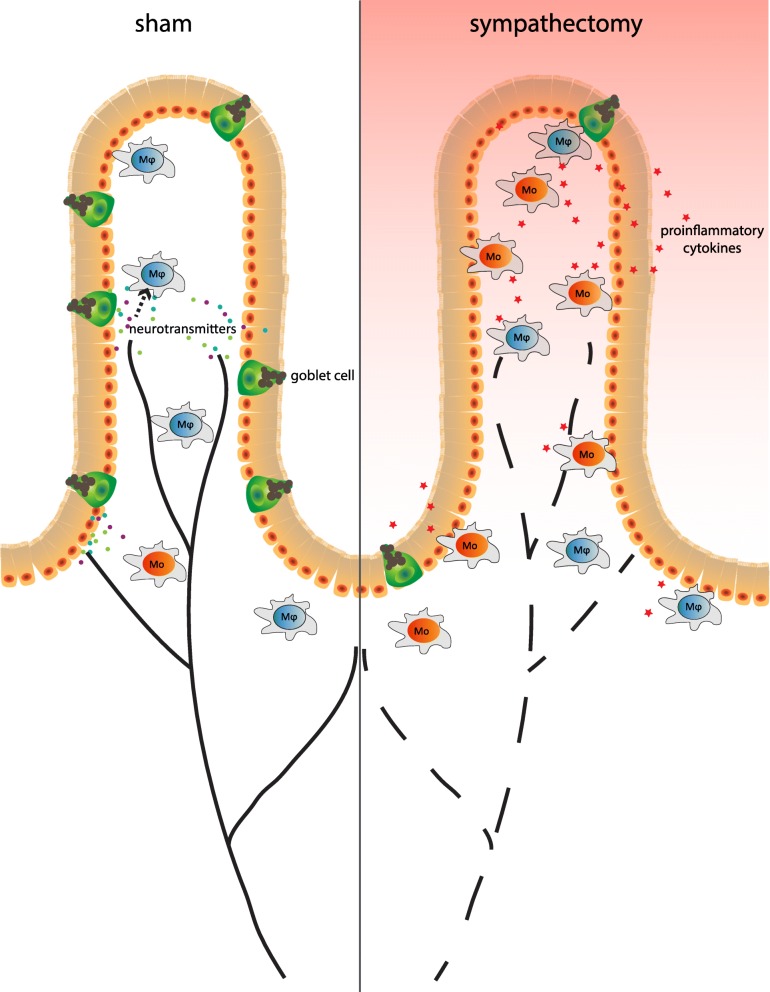
Hypothesized model. Schematic overview of the colon before and after sympathetic denervation. In Rag1^−/−^ mice after sympathectomy, there are more immature monocytes (‘Mo’, in red) compared to mature macrophages (‘Mϕ’, in blue), there is goblet cell depletion and levels of pro-inflammatory cytokines are higher compared to the sham group

Here, we describe colitis upon sympathectomy only seen in Rag1^−/−^ mice since we confirmed in an earlier report that wild-type mice, C57Bl/6, do not develop colitis upon sympathectomy (Willemze et al. 2018). This is likely due to T-lymphocytes that exert important regulatory functions that control macrophage polarization by expressing cytokines like IFN-γ, IL-4 and IL-10 (Biswas and Mantovani 2010; Munder et al. 1998; Munder et al. 1999). If T-lymphocytes and thus these signals are absent, myeloid cells likely need a critical level of sympathetic input to maintain immune homeostasis in the gut mucosa where inappropriate immune responses to commensal bacteria need to be reduced (Fig. 7). In addition to this, previous research has shown that the sympathetic nervous system also interacts with the lymphocyte compartment and it is already known for a long time that lymphocytes express adrenergic β2 receptors (Williams et al. 1976). We showed that T-lymphocytes in the intestine respond to adrenergic receptor signalling and express acetylcholine (Dhawan et al. 2016). Another study showed that adrenergic receptor signalling regulates lymphocyte egress from lymph nodes (Nakai et al. 2014). Thus the anti-inflammatory effect of the sympathetic nervous system in a physiological setting functions in two ways interacting with both the myeloid as well as the lymphoid compartment, highlighting the elegance and complexity of this neuro-immune interaction.

Most of the work discussed, our work included, concerns mouse data and the next critical step would be to further investigate effects of the sympathetic nervous system on the mucosal immune compartment in the human setting. It would be highly interesting to investigate the capability of human intestinal macrophages and lymphocytes to respond to norepinephrine. Our data suggests that locally activating adrenergic β2 receptors in the intestine with agonists or nerve stimulation might be beneficial for patients with inflammatory bowel disease. In addition to this it is known that catecholamine levels in the intestine of patients with IBD is decreased (Magro et al. 2002).

## Conclusions

Our data indicate that, in the absence of lymphocytes, intact sympathetic innervation is required for mucosal immune homeostasis. This is a step towards better understanding of the (patho)physiology of neuro-immune interaction in the context of colon inflammation, paving the way for targeted restoration of sympathetic innervation patterns inflamed colonic mucosa.

## Additional files

Additional file 1: **Figure S1.** Lipopolysaccharide (LPS)-exposed human macrophages show a mild reduction of cytokines at protein level after pre-treatment with salbutamol. (A&C) mRNA expression levels in cell lysate of interleukin (IL)-1β, IL-6, IL-10, IL-12 and tumour necrosis factor (TNF)-α of human macrophages derived from peripheral blood after treatment with 100 ng/ml LPS for 18 h and pre-treatment with different concentrations norepinephrine (A) or salbutamol (C) together with propranolol, normalized for reference genes β-actin and β2 microglobulin. (B&D) Protein levels in supernatant of IL-1β, IL-6, IL-10, IL-12 and TNF-α of human macrophages derived from peripheral blood after treatment with 100 ng/ml LPS for 18 h and pre-treatment with different concentrations norepinephrine (B) or salbutamol (D) together with propranolol. Expression and protein levels are normalized per donor to LPS-exposed macrophages without pre-treatment since cytokine levels of LPS-untreated macrophages was often not detectable. *N* = 3 human buffy coats. Data is expressed as median and individual data points. We tested for statistical significant differences with a Kruskal-Wallis test and post-hoc Dunn’s test. *P*-value < 0.05 was considered significant. * P-value < 0.05. (PDF 138 kb)

Additional file 2: **Figure S2.** Haematoxylin and eosin (HE) stainings of the colon showing examples of subscores found compared Rag1^−/−^ after sham laparotomy or sympathectomy. (A) The left pictures represents no infiltration of leukocytes, score 0. The middle picture represents a score 1, infiltration in the mucosa (indicated with an asterisk). The right picture represents a score 3, infiltration in the muscularis (indicated with an asterisk). 10x magnification (B) The areas within the red boxes show goblet cell depletion. The middle picture represents a score 1 (less than 10%), and the right picture is an example of a score 2 (10–50%). An enlargement of goblet cell depletion is shown of the right picture. 5x magnification. (C) Asterisk shows crypt loss. In this experiment no score higher than 1 (less than 10%) was found. 5x magnification. (D) Asterisk shows a crypt abscess (in the crypt left of the asterisk). 10x magnification (E) This picture shows an example of an ulcer. 10x magnification. (F) Epithelial crypt length, indicated with a red arrow. The left picture represents no epithelial hyperplasia, score 0. The middle picture represents a score of 1 (slight hyperplasia), indicated with an asterisk. The right picture represents subscore 2 of hyperplasia (2-3x increase of crypt length). 10x magnification. (PDF 99867 kb)

Additional file 3: **Figure S3.** Adrenergic β2 receptor activation reduced lipopolysaccharide (LPS)-induced inflammatory responses in macrophages derived from Rag1^−/−^ mice. (A) mRNA expression levels of interleukin (IL)-1β, IL-6, IL-10, IL-12 and tumour necrosis factor (TNF)-α in cell lysate of BMDM from Rag1^−/−^ littermate controls of Rag1^−/−^Adrβ2^−/−^ mice after treatment with 100 ng/ml LPS for 18 h and pre-treatment with 10 μM norepinephrine or 10 μM salbutamol. (B) mRNA expression levels of IL-1β, IL-6, IL-10, IL-12 and TNF-α in cell lysate of BMDM from Rag1^−/−^Adrβ2^−/−^ mice after treatment with 100 ng/ml LPS for 18 h and pre-treatment with 10 μM norepinephrine or 10 μM salbutamol. We normalized expression for reference genes glyceraldehyde-3-phosphate dehydrogenase (GAPDH) and ribosomal protein, large, P0 (RPLP0). Expression is relative to mRNA expression in LPS-stimulated BMDM. *N* = 4 mice. Data is expressed as median and individual data points. We tested for statistical significant differences with a Kruskal-Wallis test and post-hoc Dunn’s test. *P*-value < 0.05 was considered significant. *P-value < 0.05. (PDF 136 kb)

## Abbreviations

6-OHDA: 6-hydroxydopamine
Arg1: Arginase 1
BMDM: Bone marrow derived macrophages
DefA: Defensin α
ECAR: Extracellular Acidification Rate
FCCP: Trifluoromethoxy carbonylcyanide phenylhydrazone
GALT: Gut-associated lymphoid tissue
GAPDH: Glyceraldehyde-3-phosphate dehydrogenase
HE: Haematoxylin and eosin
HPRT: Hypoxanthine phosphoribosyltransferase
i.p.: Intraperitoneally
IBD: Inflammatory bowel disease
IFN: Interferon
IL: Interleukin
IQR: Interquartile range
LPS: Lipopolysaccharide
Muc2: Mucin 2
Nos2: Nitric oxide synthase 2
OCR: Oxygen Consumption Rate
Reg3γ: Regenerating islet-derived protein 3 γ
SD: Standard deviation
TNF: Tumour necrosis factor
