# Supplementary figures and images for: Loss of intestinal sympathetic innervation elicits an innate immune driven colitis
Source: Mol Med. 2019 Jan 7;25:1. doi: 10.1186/s10020-018-0068-8 (PMC6322236; doi:10.1186/s10020-018-0068-8)

Suppl. Figure 1

A

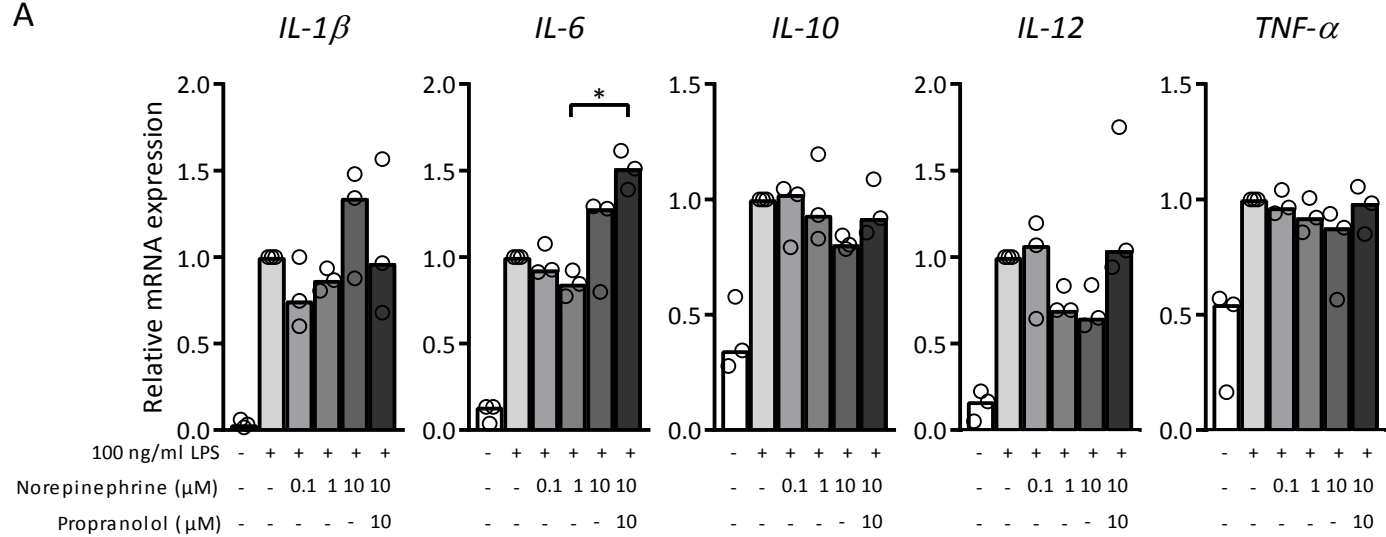

B

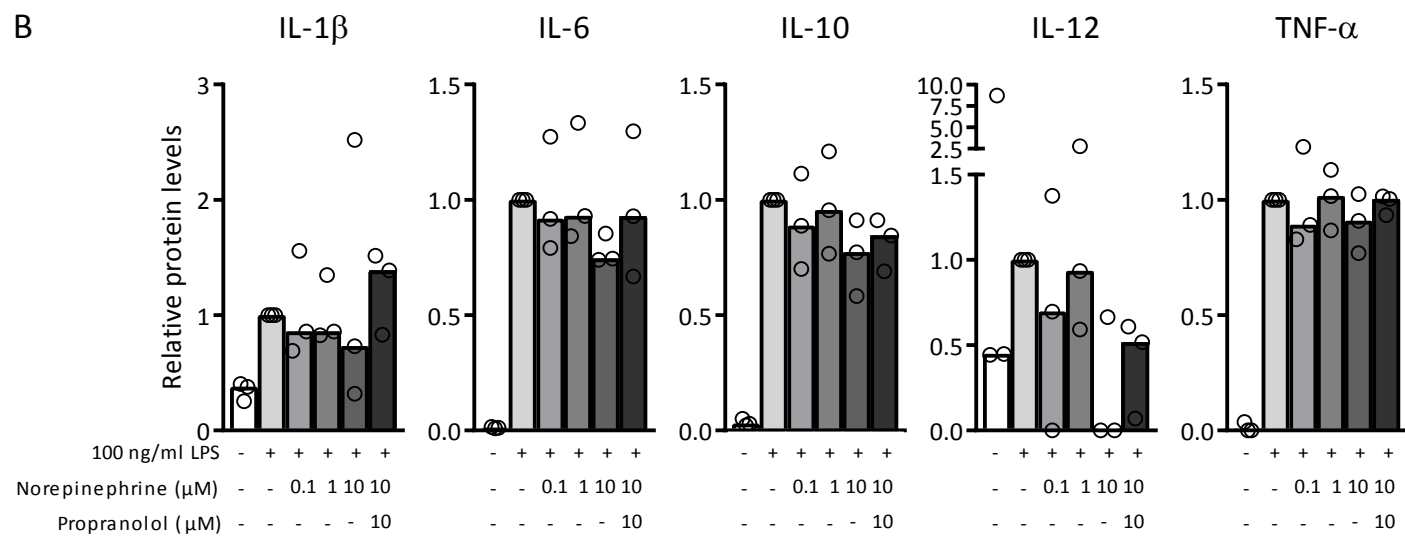

C

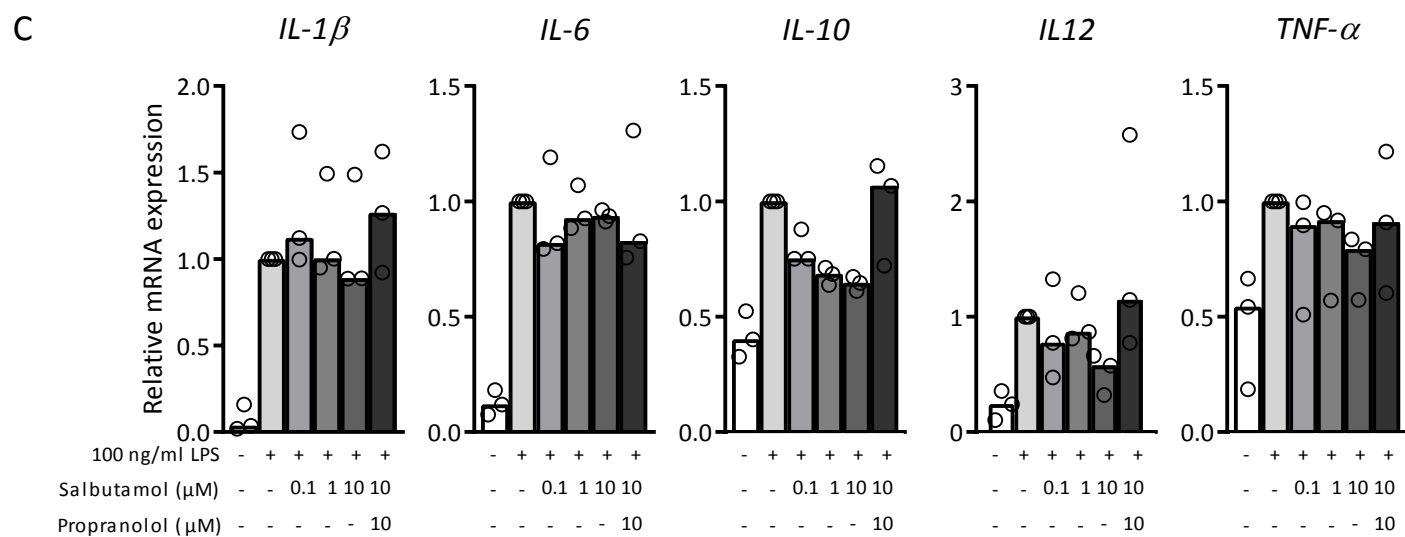

D

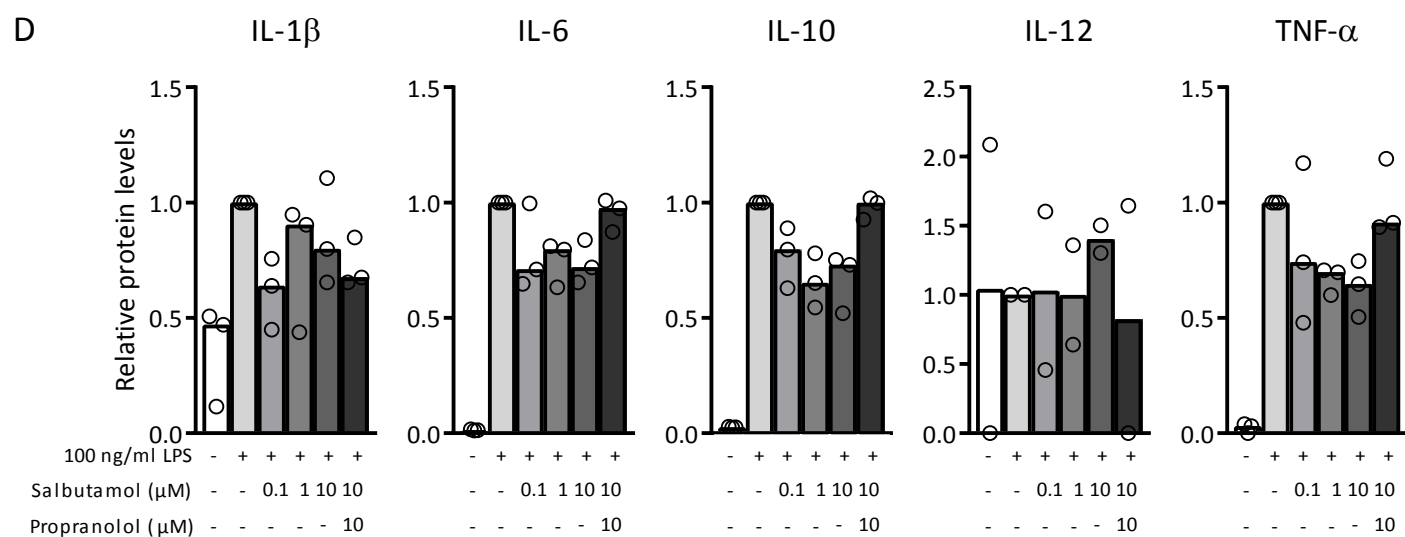

Supplement: Supplementary file 1 — Figure S1. Lipopolysaccharide (LPS)-exposed human macrophages show a mild reduction of cytokines at protein level after pre-treatment with salbutamol. (A&C) mRNA expression levels in cell lysate of interleukin (IL)-1β, IL-6, IL-10, IL-12 and tumour necrosis factor (TNF)-α of human macrophages derived from peripheral blood after treatment with 100 ng/ml LPS for 18 h and pre-treatment with different concentrations norepinephrine (A) or salbutamol (C) together with propranolol, normalized for reference genes β-actin and β2 microglobulin. (B&D) Protein levels in supernatant of IL-1β, IL-6, IL-10, IL-12 and TNF-α of human macrophages derived from peripheral blood after treatment with 100 ng/ml LPS for 18 h and pre-treatment with different concentrations norepinephrine (B) or salbutamol (D) together with propranolol. Expression and protein levels are normalized per donor to LPS-exposed macrophages without pre-treatment since cytokine levels of LPS-untreated macrophages was often not detectable. N = 3 human buffy coats. Data is expressed as median and individual data points. We tested for statistical significant differences with a Kruskal-Wallis test and post-hoc Dunn’s test. P-value < 0.05 was considered significant. * P-value < 0.05. (PDF 138 kb) [file 10020_2018_68_MOESM1_ESM.pdf]

Suppl. Figure 2

A

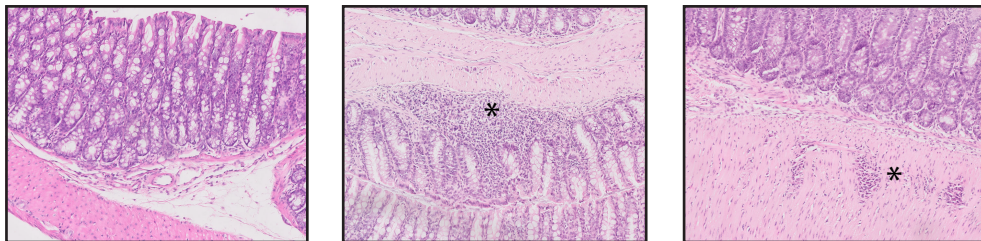

B

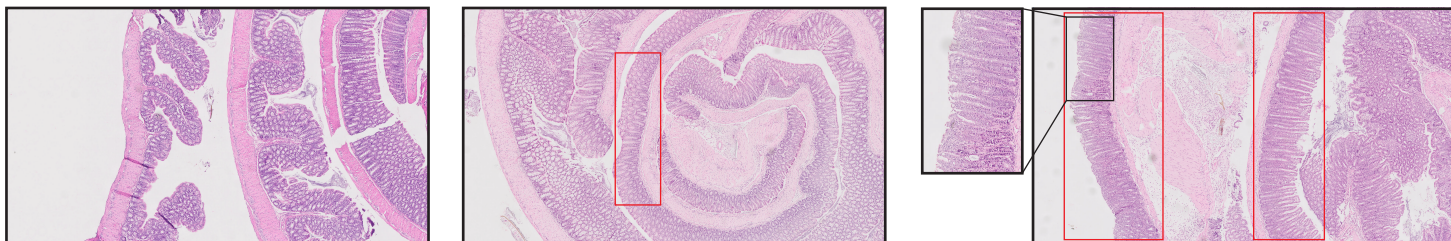

C

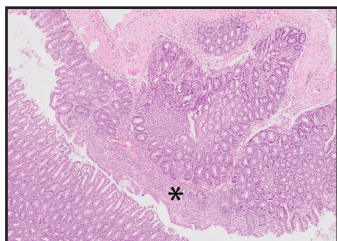

D

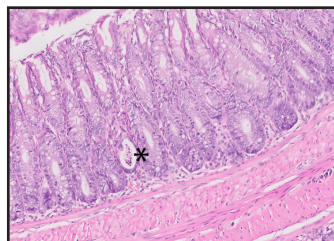

E

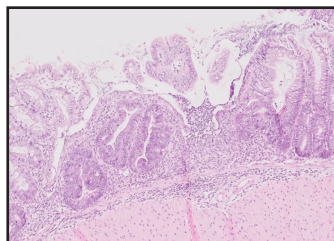

F

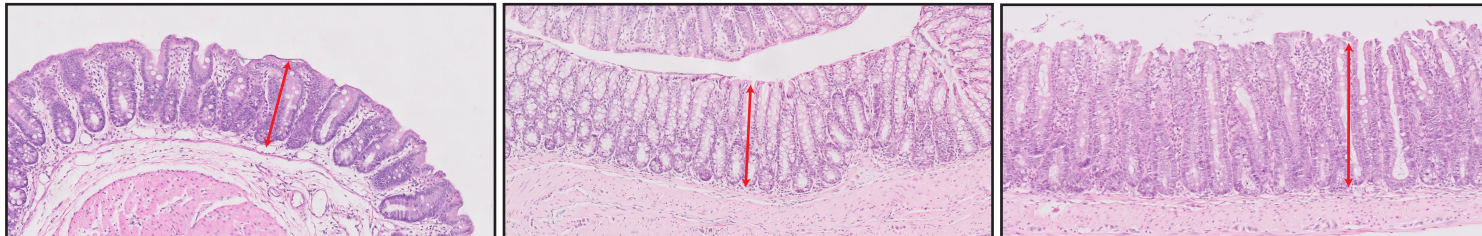

Supplement: Supplementary file 2 — Figure S2. Haematoxylin and eosin (HE) stainings of the colon showing examples of subscores found compared Rag1−/− after sham laparotomy or sympathectomy. (A) The left pictures represents no infiltration of leukocytes, score 0. The middle picture represents a score 1, infiltration in the mucosa (indicated with an asterisk). The right picture represents a score 3, infiltration in the muscularis (indicated with an asterisk). 10x magnification (B) The areas within the red boxes show goblet cell depletion. The middle picture represents a score 1 (less than 10%), and the right picture is an example of a score 2 (10–50%). An enlargement of goblet cell depletion is shown of the right picture. 5x magnification. (C) Asterisk shows crypt loss. In this experiment no score higher than 1 (less than 10%) was found. 5x magnification. (D) Asterisk shows a crypt abscess (in the crypt left of the asterisk). 10x magnification (E) This picture shows an example of an ulcer. 10x magnification. (F) Epithelial crypt length, indicated with a red arrow. The left picture represents no epithelial hyperplasia, score 0. The middle picture represents a score of 1 (slight hyperplasia), indicated with an asterisk. The right picture represents subscore 2 of hyperplasia (2-3x increase of crypt length). 10x magnification. (PDF 99867 kb) [file 10020_2018_68_MOESM2_ESM.pdf]

A

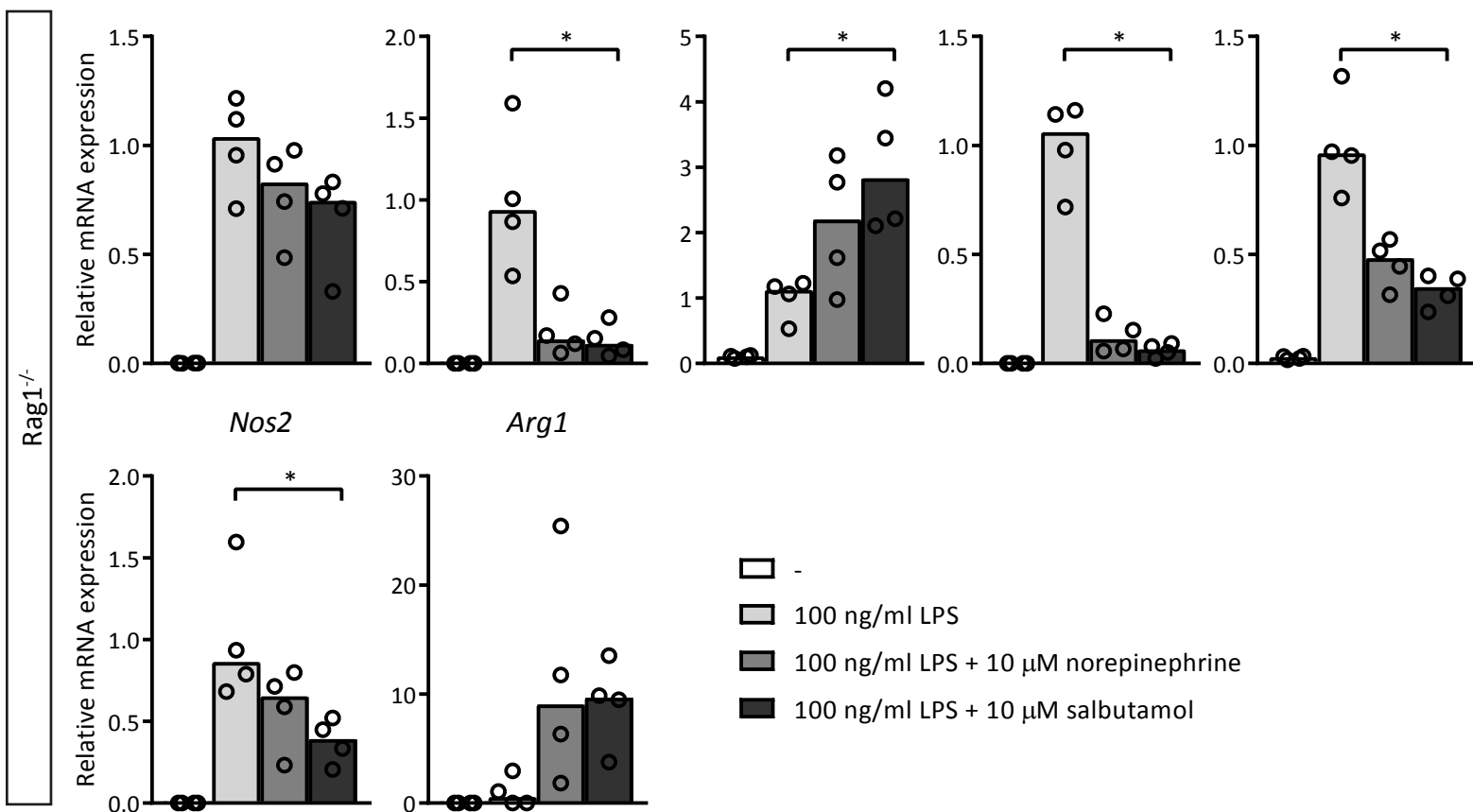

B

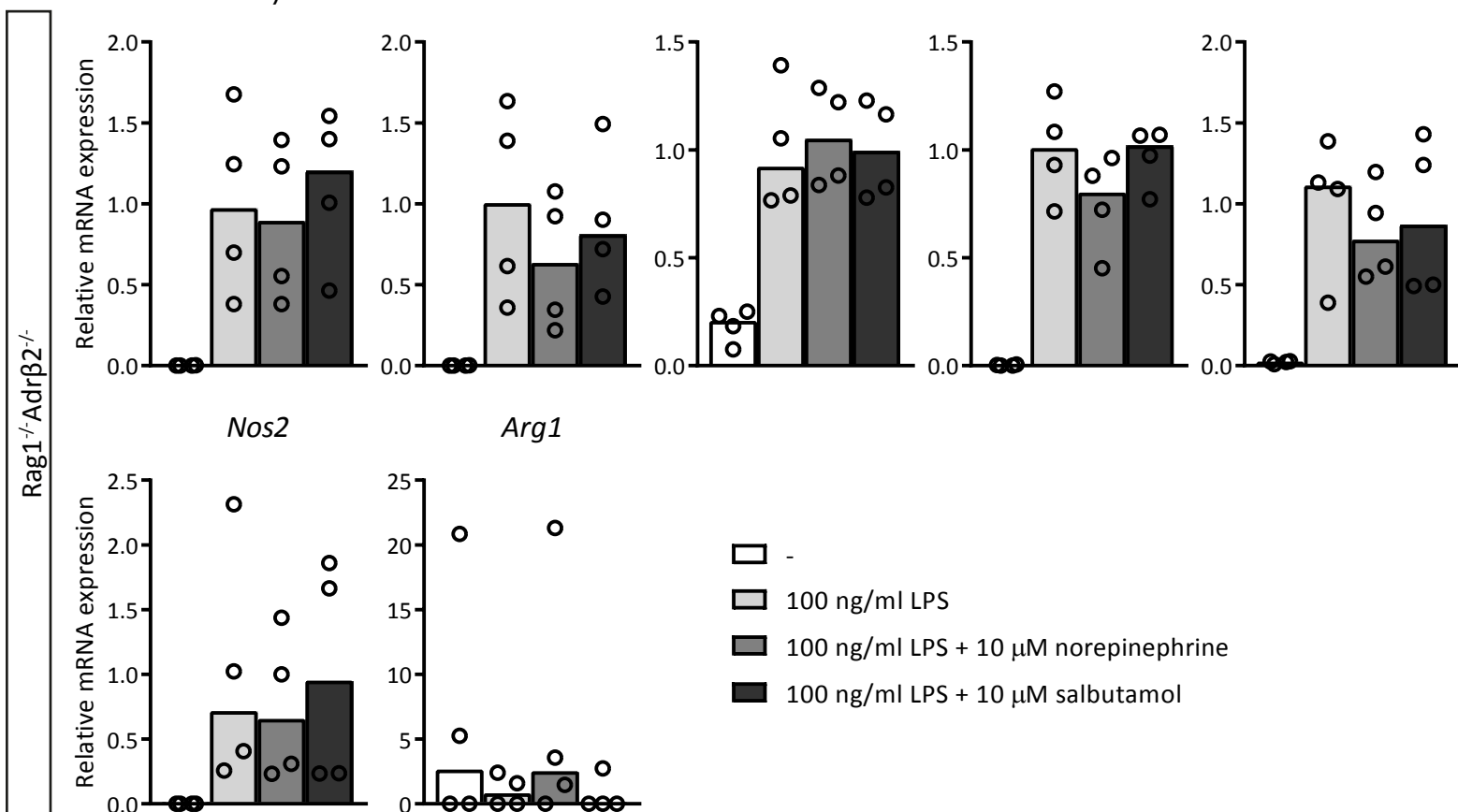

Supplement: Supplementary file 3 — Figure S3. Adrenergic β2 receptor activation reduced lipopolysaccharide (LPS)-induced inflammatory responses in macrophages derived from Rag1−/− mice. (A) mRNA expression levels of interleukin (IL)-1β, IL-6, IL-10, IL-12 and tumour necrosis factor (TNF)-α in cell lysate of BMDM from Rag1−/− littermate controls of Rag1−/−Adrβ2−/− mice after treatment with 100 ng/ml LPS for 18 h and pre-treatment with 10 μM norepinephrine or 10 μM salbutamol. (B) mRNA expression levels of IL-1β, IL-6, IL-10, IL-12 and TNF-α in cell lysate of BMDM from Rag1−/−Adrβ2−/− mice after treatment with 100 ng/ml LPS for 18 h and pre-treatment with 10 μM norepinephrine or 10 μM salbutamol. We normalized expression for reference genes glyceraldehyde-3-phosphate dehydrogenase (GAPDH) and ribosomal protein, large, P0 (RPLP0). Expression is relative to mRNA expression in LPS-stimulated BMDM. N = 4 mice. Data is expressed as median and individual data points. We tested for statistical significant differences with a Kruskal-Wallis test and post-hoc Dunn’s test. P-value < 0.05 was considered significant. *P-value < 0.05. (PDF 136 kb) [file 10020_2018_68_MOESM3_ESM.pdf]
